# Retrieving 3D medical data along fitted curved slices and their display

**DOI:** 10.1186/s12911-019-1018-2

**Published:** 2020-02-07

**Authors:** Marco Paluszny, Dany Ríos

**Affiliations:** Universidad Nacional de Colombia, Carrera 65, No. 59a, Medellín, Colombia

**Keywords:** Computed tomography, Magnetic resonance, Developable surface, DICOM

## Abstract

**Background:**

Computeros tomography and magnetic resonance imaging are usually offered to the clinician in the form of sequences of axial, sagittal and coronal planar cuts. Frequently this does not allow for the full inspection of the morphology of the area of interest, because it is limited by the planarity. Efforts have been made to extract information along curved slices but their planar display is prone to metric deformation.

**Methods:**

We propose a new visualization alternative of 3*D* medical volumes using curved slices adapted to areas of interest. We use surfaces fitted to specific organs as visualization canvasses. We describe the differential geometry techniques used to build the surfaces that may be isometrically flattened. These are referred to as develpable surfaces.

**Results:**

We show concrete examples deemed useful for the development of clinical and educational tools. Our examples are centered in magnetic resonance data of the rotator cuff muscle complex and computed tomography data of maxillofacial and dental studies. We also look at the extraction and display of information from volumes of aortic aneurysms along transversal surfaces.

**Discussion:**

We look at extensions of the technique and propose further possible clinical use of texturized surfaces in the context of volume navigation.

**Conclusions:**

We presented a technique to extract information from computer tomography and magnetic resonance volumes, using two different texturization techniques. In the cases that the fitting surfaces are chosen to be developable, they may be flattened without distortion. We also discuss how tu use the technique in other visualization tasks such as volume navigation and detection of volumetric features.

## Background

The adoption of imaging techniques to inspect 3*D* body organs and clinically significant structures has been increasingly present in many medical and odontological areas. Already in 1989, the visionary work of Fuchs, Levoy and Pizer [1] points to the need of computer graphics techniques in medical imaging applications. The review of Zhang, Eagleson and Peters [2] sketches 2*D* and 3*D* visualization techniques in medicine. The recent work of Wheeler et al. [3] shows that it is feasible to build a 3*D* interactive environments to inspect medical volumes, but it comes at a high computational cost. In fact, in the last twenty years the volume of 3*D* medical data has been growing significantly [4], and this is being compounded with upward trends in archiving, sharing, and data availability. This provides a huge opportunity for the geometry community for development and testing of technologies for the visualization of medical volumes. One of the enabling factors is the existence of universally accepted dicom standard, which provides fairly structured reporting and allows for imaging study review with publicly available software^1^. In particular, Computed Tomography (CT) and Magnetic Resonance Imaging (MRI) are standard tools for internal organ visualization in dentistry, and in medical practice in general. But, if no sophisticated visualization software is available the CT or MRI information used for diagnosis is usually restricted to flat slices that can only be inspected sequentially 2.

**Fig. 1:**
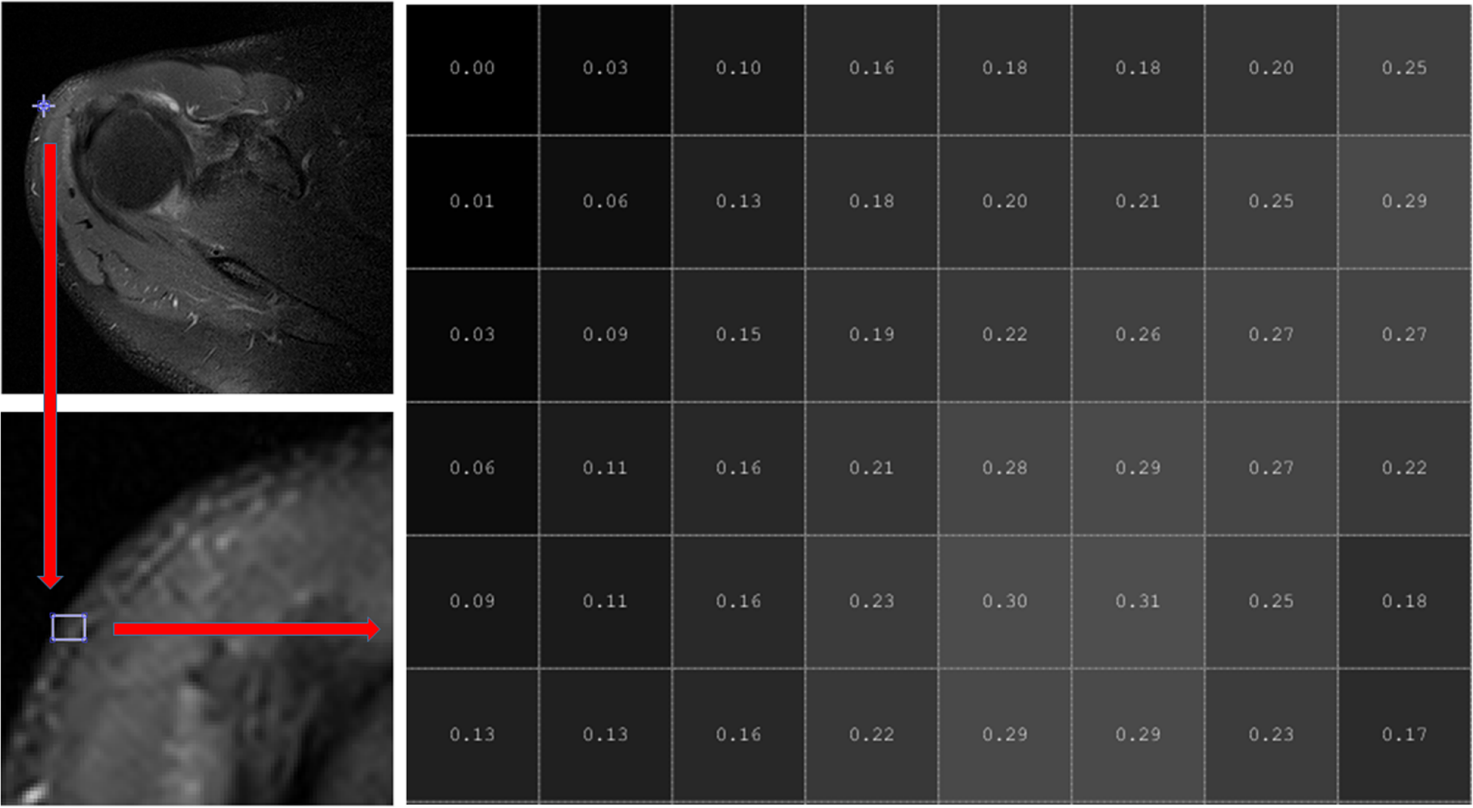
Pixel values of a region of one slice of a rotator cuff MRI

**Fig. 2:**
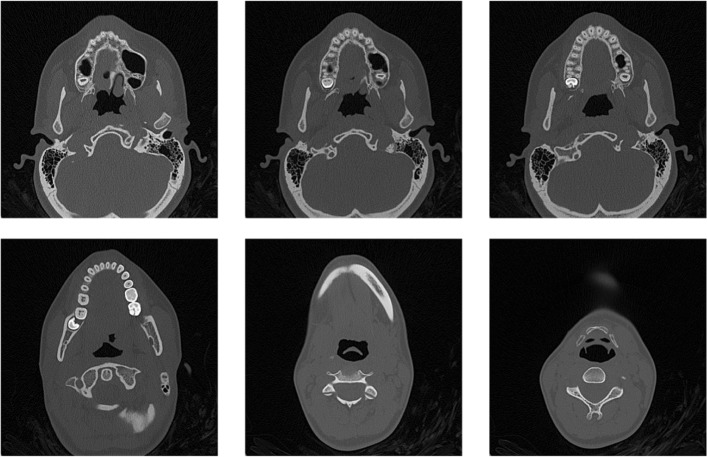
Six axial dental slices, not all consecutive. Images are 512×512 pixels

Any image to be inspected arises by setting each pixel to the gray level corresponding to the tissue type at its position in 3*D*, and this is referred to as the texturization process, see Fig. 1. The full set (usually ranging from under a hundred to a few hundred) of these planar images is referred to as a CT (or MRI) volume. Figure 2 shows some slices of a dental maxillofacial CT.

**Fig. 3:**
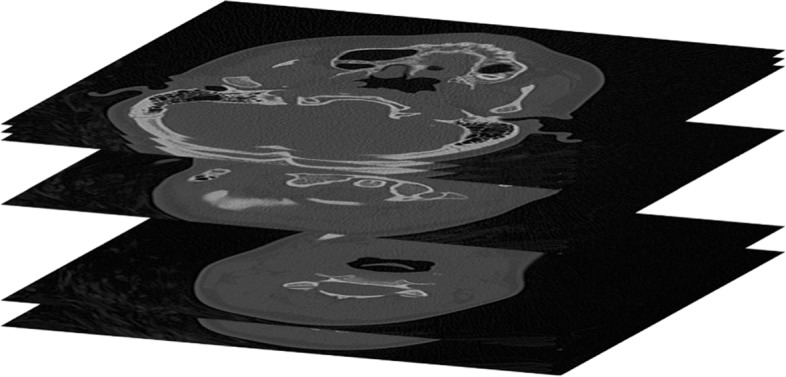
Positions in space of slices of Fig. 2

**Fig. 4:**
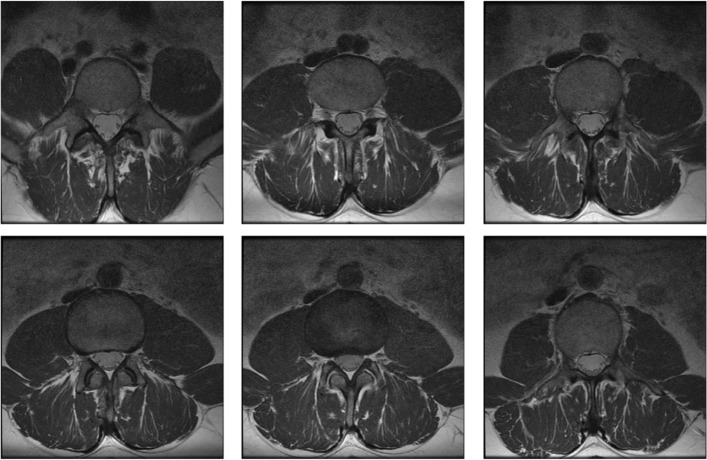
Six not parallel slices of the stomach

**Fig. 5:**
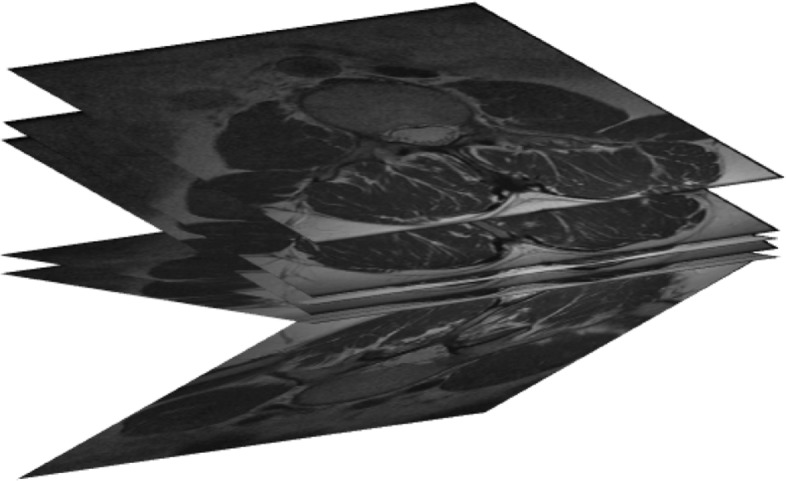
Positions in space of the stomach slices of Fig. 4

Usually the set of planar slices conforming a medical volume are parallel in 3*D* and live in a rectangular box. Figure 3 illustrates the placement in 3*D* of the planar slices of Fig. 2. Note that the slices do not have to be equidistant in space. For some organs, it may happen, that the actual positions of the slices in 3*D* space are not parallel in 3*D*. For, an example of six stomach slices and their actual positions in space is illustrated in Figs. 4 and 5.

## Methods

DICOM (Digital Imaging and Communications in Medicine) is a communications protocol and file format to store medical information from imaging studies. Each.dcm file contains the gray levels of one slice of a body part. The standard software (which is usually provided by the vendor together with the dicom files of the study) allows for the display of the planar slices as shown in Figs. 2 and 4.

**Fig. 6:**
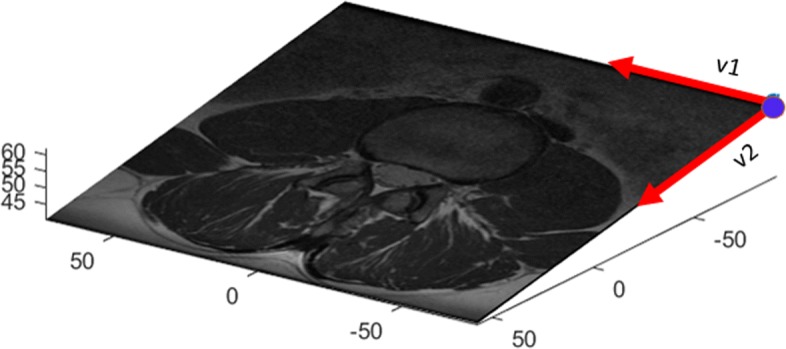
Reference origin and generating vectors of the 3*D* plane containing a stomach slice

Each.dcm file has a header with patient information such as age, gender, height, the scanner characteristics and scanning parameters. The header also contains the geometry information that allows for the precise positioning of the data points in 3*D* and their gray level. The corresponding fields are ImagePositionPatient, ImageOrientationPatient and PixelSpacing^3^. Figure 6 illustrates the origin and the axes of the plane containing the texturized rectangle corresponding to a.dcm file. Figures 3 and 5 illustrate the (position) of the slices of two studies.

Real organs in 3D usually do not conform to planar slices. For example the human maxillofacial bone tends to follow a parabola [5]. Recently there have been efforts to explore MRI and CT volumes along non planar slices [6–8]. In the next subsection we consider extracting information from 3*D* volumes using curved slices which adapt to the organs to be inspected.

### Painting the curved slices within a 3D volume

The sequence of dicom files determine a volume, usually a box^4^

$$\{ (x,y,z):x_{0}< x< x_{1}, y_{0}< y< y_{1}, z_{0}< z< z_{1}\} $$

where *x*, *y* and *z* are the standard cartesian coordinates and *x*_*i*_, *y*_*i*_ and *z*_*i*_ give the extensions of the inspected volume. The information necessary to determine these parameters is provided in the following fields of the dicom files: ImagePositionPatient, ImageOrientationPatient and PixelSpacing. The box has a natural discretization which, most of the time, may be made to coincide with the positions of the data points in the volume, as prescribed by the fields in the headers. Hence each point of the discretization may be assigned a gray level provided by the dicom file. Given a curved slice within the volume above, to each of its points we assign a gray level according to the gray levels of the nearest points in the discretization. This process is referred to as texturization and it is amply used in computer graphics to “paint” surfaces, [9].

There are various texturization techniques, two possibilities are: trilinear interpolation and nearest neighbour approximation.

### Trilinear interpolation

Trilinear interpolation is a widely used technique, see [10, 11], in computer art and virtual reality, and in general, to display computer generated 3D objects. It is a mathematical algorithm that assigns a color (or a level of gray) to a point in 3*D* space based on the colors (or levels of gray) at the vertices of the box that contains it.

More precisely, trilinear interpolation involves three linear interpolations, namely, given two points **a** and **b** in 3*D* for any point **x** on the segment joining them, there is *λ*∈[0,1] such that **x**=(1−*λ*)**a**+*λ***b**.

Let $\mathbf {a}_{_{FL}},\mathbf {a}_{_{FR}},\mathbf {a}_{_{BL}},\mathbf {a}_{_{BR}}$ and $\mathbf {b}_{_{FL}},\mathbf {b}_{_{FR}},\mathbf {b}_{_{BL}},\mathbf {b}_{_{BR}}$ be the vertices of a box^5^, then any **x** inside the box may be written

1 $$  \begin{array}{ll} \mathbf{x} =& (1-\alpha)(1-\beta)[(1-\gamma)\mathbf{a}_{_{FL}}+\gamma\mathbf{a}_{_{FR}}]\\&+(1-\alpha)\beta[(1-\gamma)\mathbf{a}_{_{BL}}+\gamma\mathbf{a}_{_{BR}}] \\ &+\alpha(1-\beta)[(1-\gamma)\mathbf{b}_{_{FL}}+\gamma\mathbf{b}_{_{FR}}] \\&+\alpha\beta[(1-\gamma)\mathbf{b}_{_{BL}}+\gamma\mathbf{b}_{_{BR}}] \end{array}  $$

for some *α*,*β* and *γ* in [0,1].

In the case that the medical volume is built from dicom files corresponding to rectangles positioned on parallel planes in 3*D*, arranged in a box, each rectangle is discretized according to the dicom headers^6^ and the 3*D* positions of these discrete points partition the volume into sub-boxes. So, any point inside the volume, but otherwise of arbitrary coordinates, is contained in one and only one sub-box and hence it may expressed as in Eq. 1 for some *α*,*β* and *γ*. The gray level assigned to **x** is given by Eq. 1 with the vertices substituted by their corresponding gray levels.

This process is used to assign a gray level to each point of the curved slice, and hence, for its painting.

### Nearest neighbor texturization technique

**Fig. 7:**
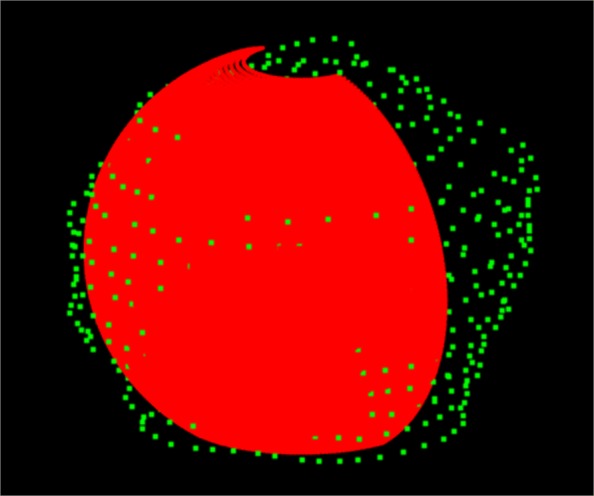
Green: Points on the humerus head surface - Red: Fitting ellipsoid
- Partition the volume bounding the set of slices in 3*D* into sub-boxes. This subdivision does not rely upon the orderly distribution of the data points in the volume, on it conforming to be a box or on the slices to be parallel.
- Each point of the curved slice’s discretization belongs to some sub-box so we assign to it the gray level of the nearest data point of that sub-box^7^.

Another painting technique, which takes more fully into account the gray level in the vecinity of a point in the curvilinear slice, as opposed to interpolating it from near points is referred to as the nearest neighbor approximation. We will discuss a slightly modified version of it which takes into account a prescribed discretization of the curved slice. It consists of the following steps:

- Choose a discretization of the curved slice given by the parametric surface, i.e. the map [0,1]×[0,1]→*S*, where *S* fits the object of interest. In Fig. 7*S* is the half ellipsoid fitting the rotator cuff muscle complex. The position of *S* is informed by the humerus head (green points). The discretization of *S* is induced by the discretization of [0,1]×[0,1] via the above map, and we have to assign a gray level to each point of the discretization of *S*.

**Fig. 8:**
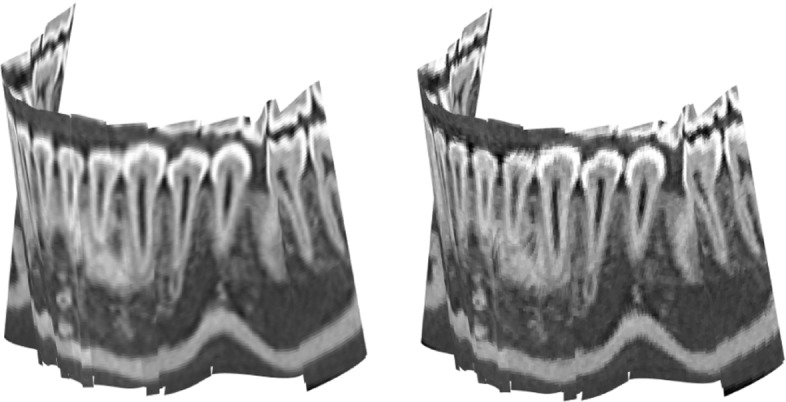
Left: Curved slice texturized by trilinear interpolation using 511×511×165=43.084.965 data points. Right: Same curved slice texturized by nearest neighbor using 20×20×20=8.000 sub-boxes

Each one of these two techniques, has particular advantages regarding the information that is retrieved along the curved slice. Trilinear interpolation tends to produce smoother displays and nearest neighbor approximation is very faithful in terms of the gray level assigned to each point since no smoothing is applied. See Fig. 8 for a comparison of a curved slice painted with trilinear interpolation and the nearest neighbor method.

## Results

We consider three visualization examples along curvilinear surfaces fitted to objects of interest:

- the humerus in order to detect tears in the rotator cuff muscle complex
- the jaw to view a curvilinear section to provide a panoramic view of all dental pieces at the same time
- the aorta to check aortic aneurysm status

### MRI: Detecting tears near the humerus head

The muscle and tendon complex surrounding the humerus head is prone to sport injuries and age related degenerations, which usually show up as tears. Shoulder MRI is a standard procedure to resolve the position and extent of the tear and it guides the clinician regarding the choice of treatment.

**Fig. 9:**
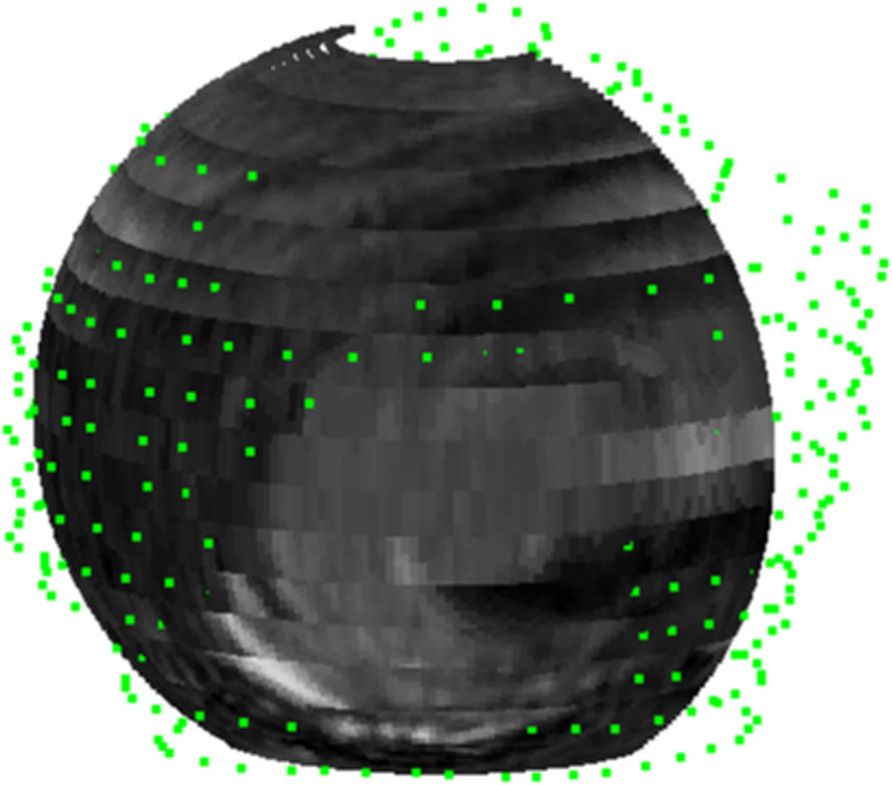
Curved slice texturized by nearest neighbor using 17^3^=4.913 sub-boxes

**Fig. 10:**
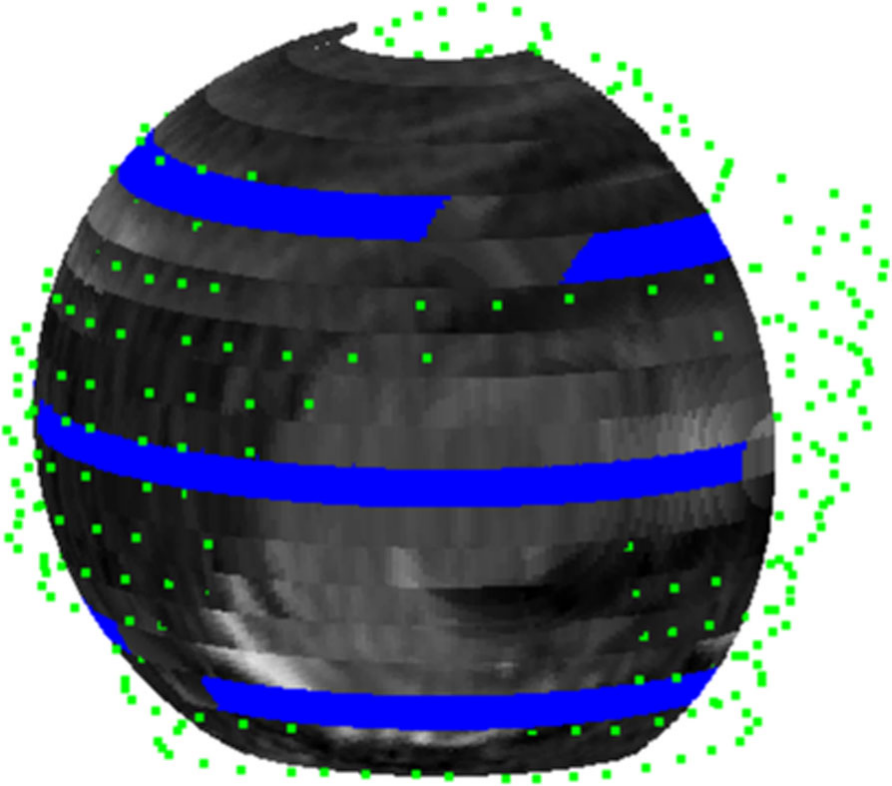
Blue areas of the curved slice correspond to points which lie in sub-boxes for which there is not DICOM provided information

Figure 9 illustrates the texturization of the fitting ellipsoid in Fig. 7 assigning gray levels to the points of its discretization. It uses the nearest neighbor technique presented in the previous section, whose salient feature is the possibility to choose the number of sub-boxes. In fact, as the number of sub-boxes increases the number of empty boxes and hence the number of uninformed points of the ellipsoid discretization also grows. Figure 9 depicts the texturization for 17^3^=4.913 sub-boxes and Fig. 10 shows the same ellipsoid’s texturization when the number of sub-boxes have been increased to 20^3^.

The two renderings are complementary, the 17^3^ sub-box rendering shows the approximate position of the tear (in our case infraespinatus and teres minor) on the fitting ellipsoid and the 20^3^ sub-box rendering pinpoints more precisely the points for which there is no dicom information.

Additionally to the tear information on the fitting ellipsoid we obtain shape and size information of the tears by considering further surfaces of revolution, namely by offsetting the circles composing the fitting ellipsoid’s circles^8^.

**Fig. 11:**
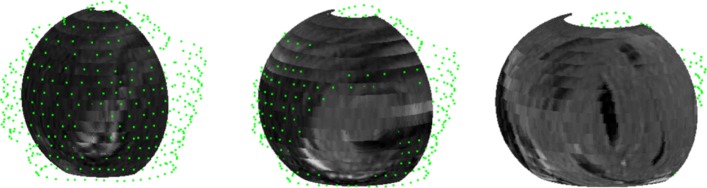
Middle: Ellipsoid of revolution fitting the humerus head. Left: Inward 4*mm*. Right: Outward 6*mm*

**Fig. 12:**
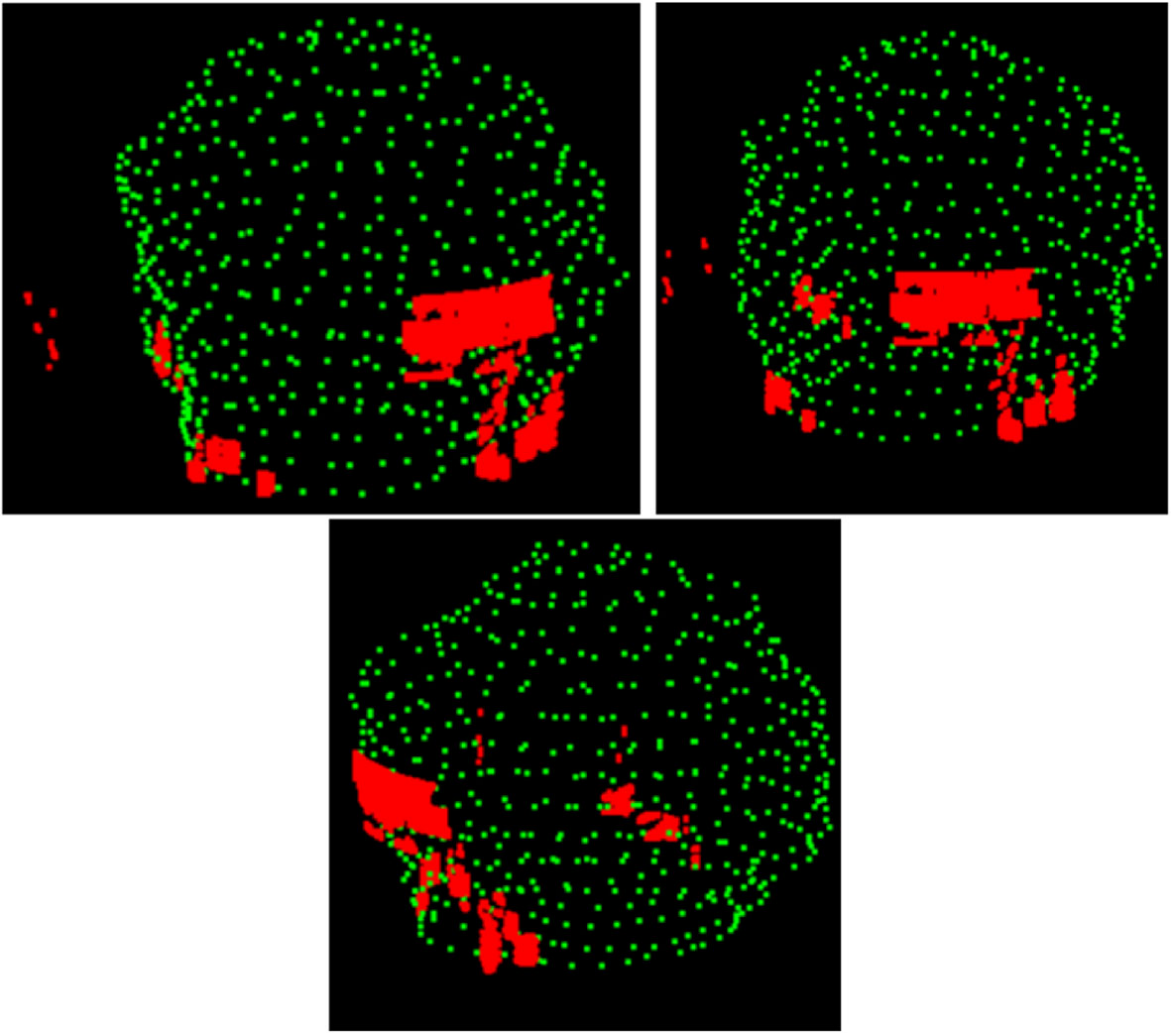
Three views of the humerus head and the tears. Green: Humerus head points. Red: Tears, Additional file 5

The MRI volume illustrated in Fig. 11 shows three curved slices, the middle one is half of an ellipsoid of revolution fitting the humerus head^9^ and two additional curved slices, one inward (radially towards the ellipsoid axis, i.e. towards the humerus bone) and the other outwards, away from the axis. Each curved slice has beeen painted with the nearest neighbor method using sixteen MRI slices and 17^3^=4.913 sub-boxes. The radial distance between the inner and the outermost curved slices in 3*D* is 10 mm. They illustrate the infraespinatus and teres minor area which show bright zones suggesting tears. Figure 12 illustrates the 3*D* rendering of the tears with the humerus head described by green points. The web version of this paper offers animations of 3*D* renderings of tears of the rotator cuff complex based on the fitting ellipsoid and several outer and inner slices. The latter are surfaces of revolution obtained by augmenting the radii of the ellipsoid circles in the normal direction to its axis.

### CT: Curved slices in dentistry

In dental applications, 3*D* visualization of individual or groups of teeth has been of increasing interest since the introduction of cone beam computed tomography (CBCT). This is specially the case in dentistry in the areas of implantology and endodontics. In implantology The American Academy of Oral and Maxillofacial Radiology, recommends CBCT for presurgical assessment of dental implant sites. In the field of endodontics in the recent book, The Guidebook to Molar Endodontics [12] the authors argue the convenience of CBCT, particularly in establishing the morphology of the area of the root system, namely the existence and 3*D* placement of isthmi and intracanal communication in molars^10^. Moreover, the utility of the knowledge of the surrounding tissues, by inspection with CBCT, has been subject of recent interest because, potentially, it has a better sensitivity and specificity than intraoral periapical radiography variations, see [13].

In this subsection we consider special types of curved slices that adapt to morphologies of interest in maxillofacial and dental imaging. Figure 8 illustrates a curved slice extracted from a tomographic volume of a human jaw. The slice follows the dental pieces allowing for their full display, together with roots, in 3*D*. Due to maxillofacial morphology the most useful types of curved slices are given by ruled surfaces. These are composed of straight line segments. A specially interesting subfamily of ruled surfaces are the developable surfaces which enjoy the property that may be flattened without metric distortion, which means that lengths and areas are exactly the same as measured on the curved slice or its flat display^11^.

Construction and texturization of curved slices described by ruled surface has been considered in [6] mainly in the case of cylinders. For another proposal for the flattened presentation of texture information along a curved slice see [14]. In odontology, [15] considered developable surfaces along Bézier curves and [8], cone splines built with circular cones.

Developable surfaces are usually built along 3*D* curves and in maxillofacial applications follow the sequence of dental pieces. There are various ways to build a developable surface that contains a prescribed curve, see [16] for a reference in the more theoretical context of Computer Aided Geometric Design and [8] for *G*^1^ conic splines built^12^ along an approximating *G*^1^ curve consisting of circle segments. The latter uses very beautiful classical geometry ideas due to Fuhs and Stachel [17].

**Fig. 13:**
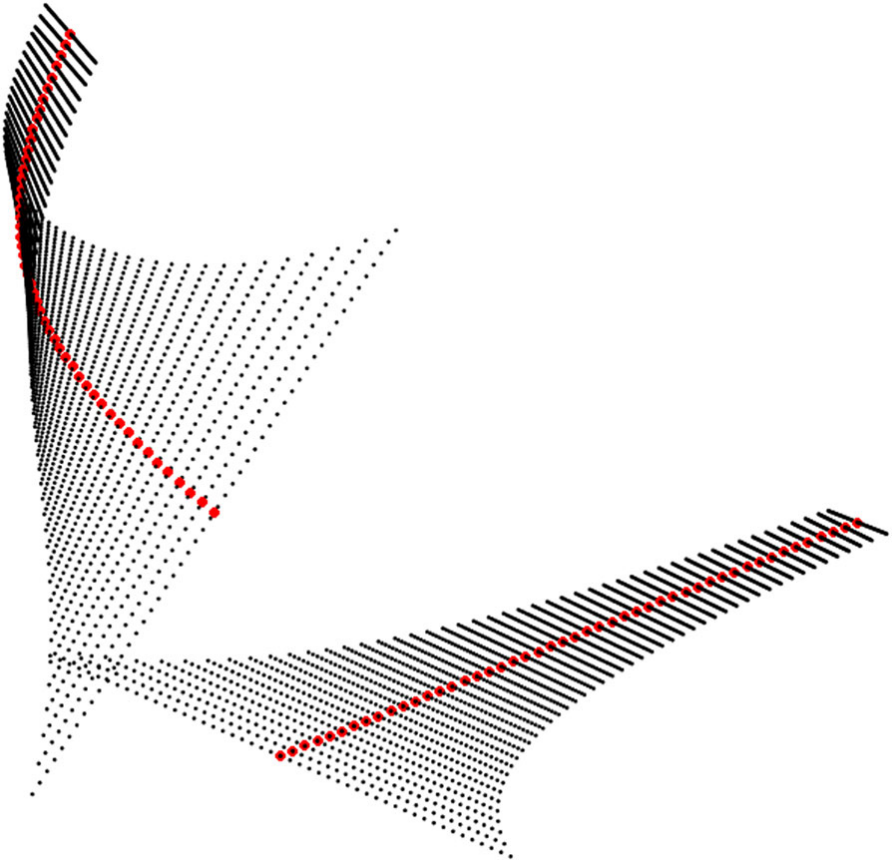
Unfolding of a developable surface along a pregeodesic (red) and its planar display, Additional file 3

A simplification occurs when the curve along which the developable surface has been built gets mapped into a straight line in the flattened image as illustrated in Fig. 13.

**Fig. 14:**
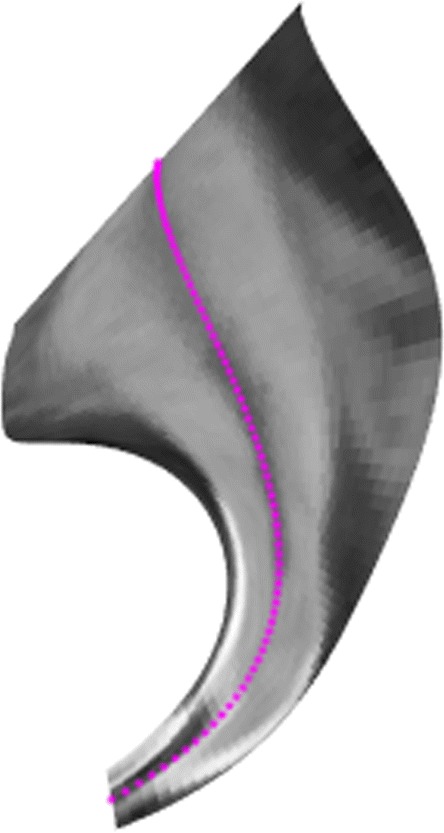
Developable surface along a space Bézier cubic that follows the root canal. We provide a video that demonstrates graphically the 3*D* shape of this developable surface in the Additional file 1 in the website version of this paper

A potentially useful application is the display of the texturized developable surface containing a root canal as displayed in Fig. 14.

**Fig. 15:**
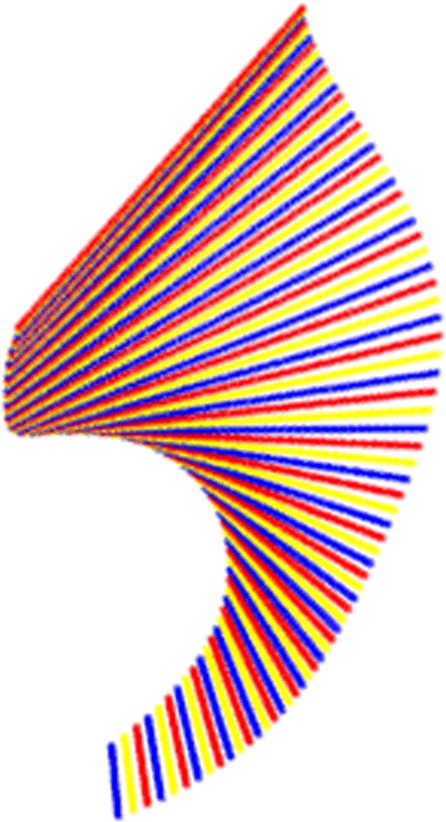
Developable surface as composed of line segments

**Fig. 16:**
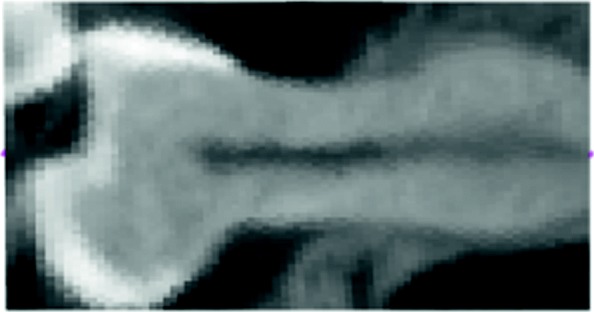
Flattened developable surface corresponding to Fig. 14

Figure 15 shows how the above texturized surface is built as a ruled surface, i.e. composed from straight line segments, and the flat (and texturized) image is presented in Fig. 16. In this particular example the 3*D* curve that approximates the root canal is mapped to a straight line segment.

**Fig. 17:**
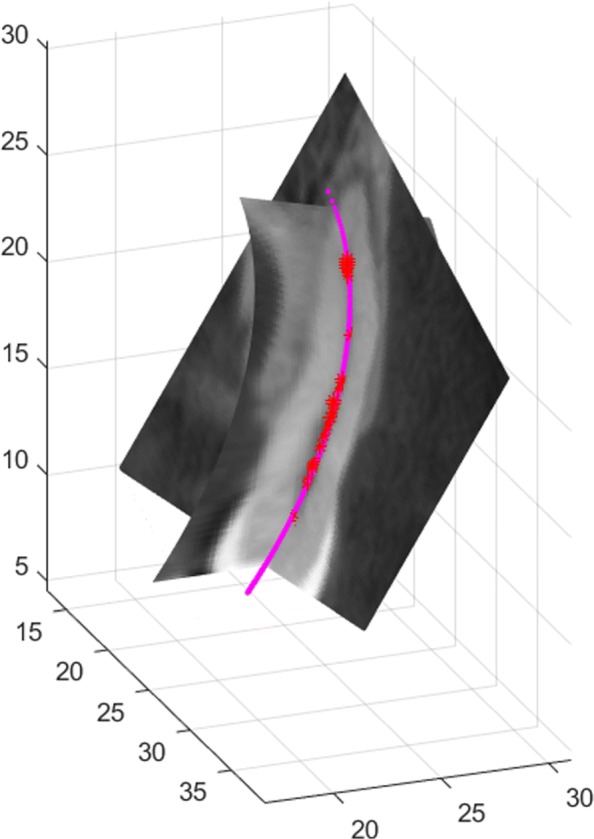
Two transversal surfaces along the root canal

Figure 17 shows two developable surfaces along the root canal allowing for the inspection of the neighboring tissues along two surfaces that intersect transversally along a canal root approximating curve.

For more information on the usefulnes of imaging in root canal treatment see [18–20].

### CT: Aortic aneurysms

An aortic aneurysm is a localized dilation of the aorta. Aneurysms rank among the leading causes of mortality in the United States. They might be asymptomatic in which case their growth should be monitored because of even higher mortality rates if rupture occurs, [21]. A recent review is [22].

**Fig. 18:**
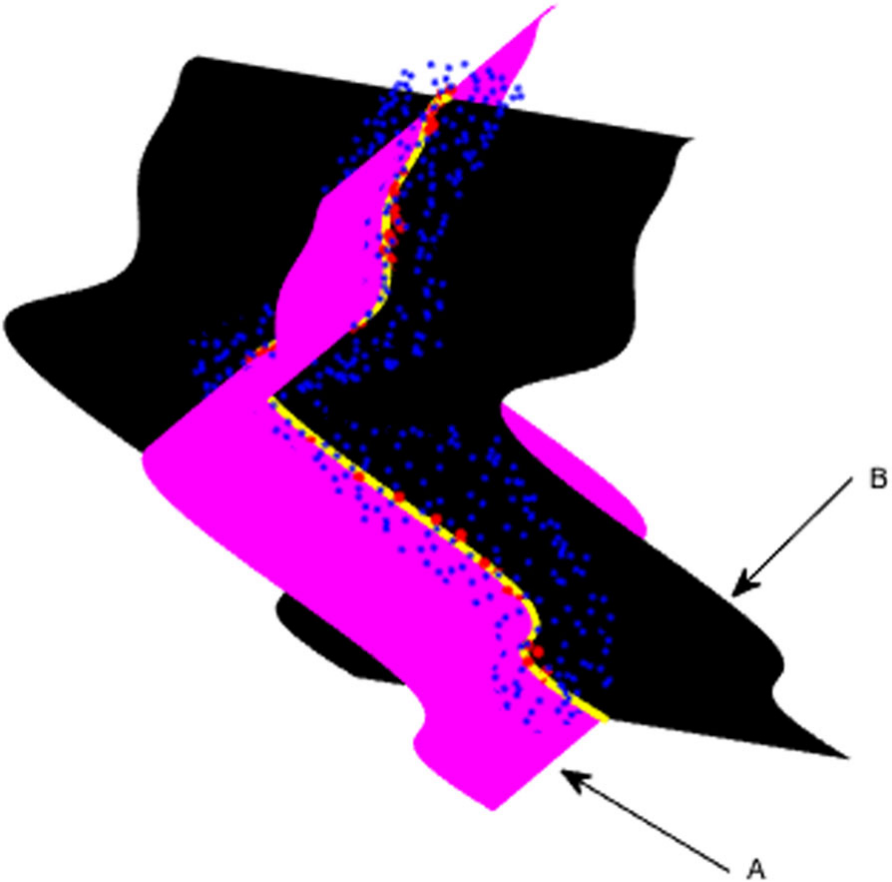
Two transversal Bézier surfaces along the center curve of an aneurysm, Additional file 2

CT scans can be used to provide information about the 3*D* geometric shape of the aneurysms by looking at its curved sections along the centerline of the aorta. Figure 18, shows two mathematical surfaces that contain the center curve of an aneurysm, and are positioned transversally with respect to each other in 3*D*. In the web version of the paper the surfaces are black and magenta, the cloud of blue points tracks the surface of the aorta, the red points sample the curve of centers of the aneurysm and the yellow line is its degree eight Bézier curve approximation. The technique utilized to create the developable surface is due to Aumann, see [16].

**Fig. 19:**
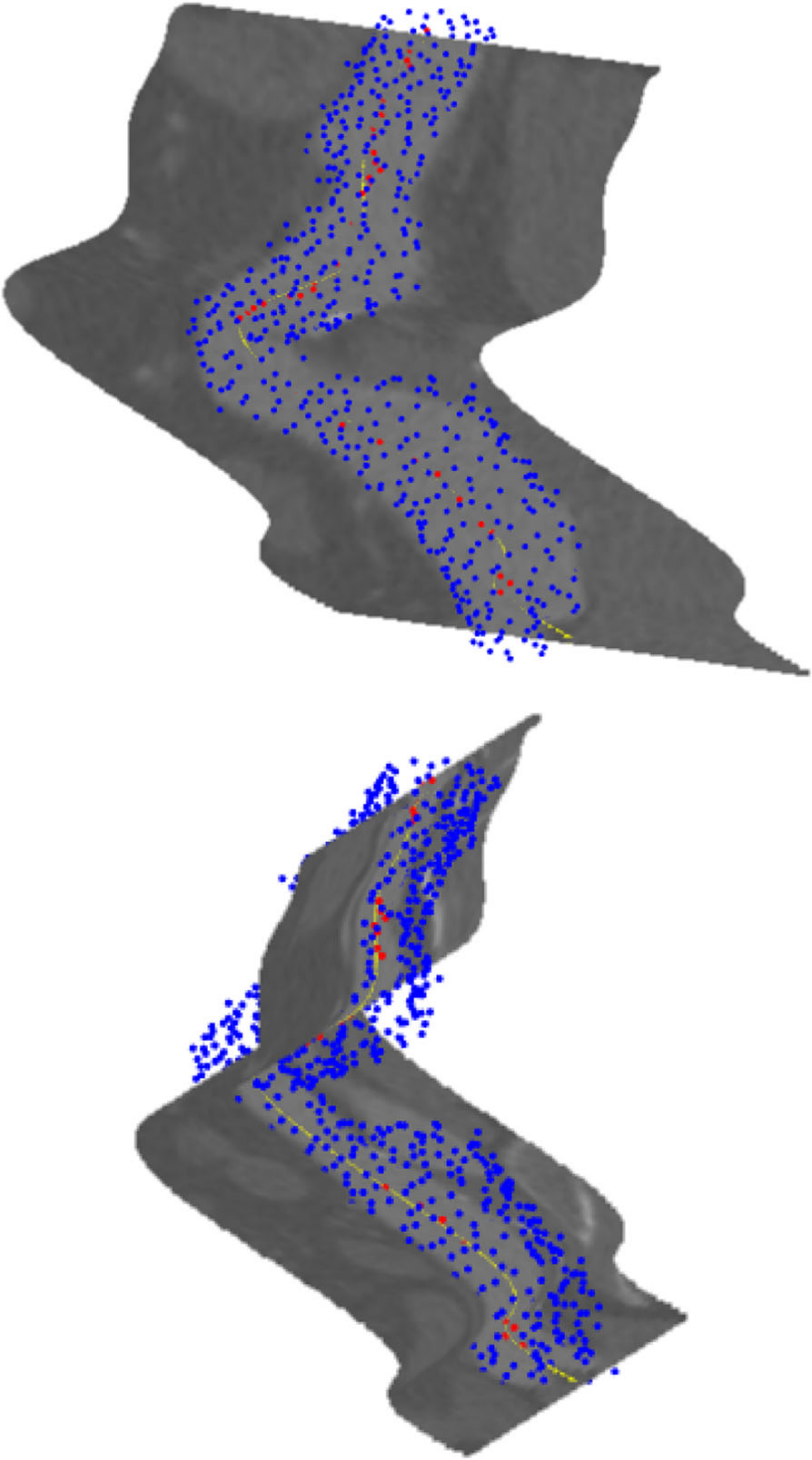
Two transversal Bézier surfaces along the center curve of an aneurysm. (Up) Fig. 18 - B and (Down) Fig. 18 - A

**Fig. 20:**
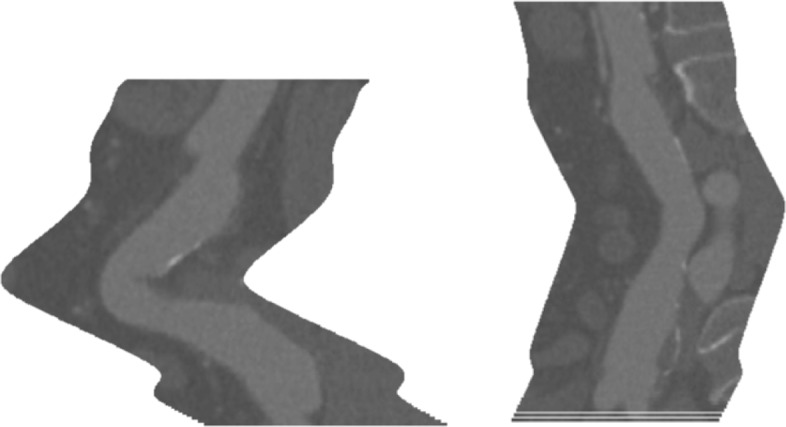
Isometrically flattened versions of the last two developable surfaces

Figure 19 shows both texturized surfaces, together with the 3*D* point cloud representing the surface of the aneurysm and Fig. 20 their isometrically flattened versions.

## Mathematical techniques

We use various types surfaces in the examples presented in this paper. In the case of aneurysms and the human jaw the parametric surface that represents a curved slice within a medical is chosen within the family of developable surfaces. These surfaces may be flattened without distortion, namely the lengths, shapes and angles are the same as measured along the 3*D* surfaces and in their flat versions. The simplest examples of developable surfaces are cones and cylinders 13.

We use two ways to build developable surfaces:

- In case of the curved slice of the jaw we use cone splines. These are built stiching together sections of cones, along common generators, such that any pair of adjacent cones share the same tangent plane along their common generator.
- For curved slices along an aneurysm we build a developable surface along a Bézier curve approximating the centerline of the aorta using Aumann’s algorithm [16]. Automatic detection of the aorta centerline has been recently discussed in [23] and the approximating Bézier curve is calculated using least squares.

To flatten without distortion a developable surface, we need to construct an explicit isometry between the surface and an open connected set in the plane, see [24]. A more efficient method, that works for any type of developable surface and does not require finding the explicit isometry, is based on the fact that the geodesic curvature of a curve on the surface does not change after isometric unfolding the surface into the plane. Namely, let *α* be a curve on the surface *S* and denote by $\bar {\alpha }$ its image under isometric unfolding of *S*. If *κ*_*g*_ is the geodesic curvature of the curve *α*^14^ on the surface *S* and $\bar {\kappa }$^15^ is the curvature of its image under isometric unfolding then $\bar {\kappa }=\kappa _{g}$, see [25]. The goal of the method is to construct a planar curve $\bar {\alpha }$ with curvature $\bar {\kappa }=\kappa _{g}$ and then build the unfolded surface by drawing the generators at the apprpriate angle to the planar curve $\bar {\alpha }$.

Recall the Frenet equations for the planar curve $\bar {\alpha }$,

$$\begin{array}{*{20}l} &\bar{e}_{1}'=\bar{\kappa}\bar{e}_{2} \\ &\bar{e}_{2}'=-\bar{\kappa}\bar{e}_{1} \end{array} $$

where $\bar {e}_{1}'$ and $\bar {e}_{2}'$ are the derivatives, with respect to arc length, of the unit tangent and the normal vector of $\bar {\alpha }$, respectively.

Applying the chain rule and considering $v(t)=\Vert \dot {\alpha }(t) \Vert = \Vert \dot {\bar {\alpha }}(t) \Vert $ we get:

$$\begin{array}{*{20}l} &\dot{\bar{e}}_{1}(t)=v(t)\kappa_{g}(t)\bar{e}_{2}(t) \\ &\dot{\bar{e}}_{2}(t)=-v(t)\kappa_{g}(t)\bar{e}_{1}(t) \end{array} $$

Since $\bar {e}_{1}$ and $\bar {e}_{2}$ are unit vectors then let $\bar {e}_{1}=[\cos (\phi (t)),\sin (\phi (t))]$ for an unknown real value function *ϕ*. Thus $\phi '(t) \bar {e}_{2}(t)=\dot {\bar {e}}_{1}(t)=v(t)\kappa _{g}(t)\bar {e}_{2}(t)$. So

$$ \phi(t)=\int v(t)\kappa_{g}(t) dt $$

Since $\bar {e}_{1}(t)$ is a unit vector, then $\dot {\bar {\alpha }}(t)=v(t)\bar {e}_{1}(t)$. Therefore the family solution of congruent curves is given by:

$$ \bar{\alpha}(t)=\int v(t)[\cos(\phi(t)),\sin(\phi(t))] dt $$

After the construction of the planar curve $\bar {\alpha }$ the final step is to construct the rulings of the unfolded surface preserving the angle enclosed by the tangent vector and the generator in 3*D*. See Fig. 13 for an illustration of this method where the red curve *α* on the surface is a geodesic, then *κ*_*g*_=0 and hence the curve $\bar {\alpha }$ is a straight line. Additional file 4 is a video of the 3D setup of the flattening method. Figure 14 shows a dental application where the magenta curve is a geodesic which interpolates the canal root of the tooth, this is why the canal root looks as a straight line in Fig. 16.

## Discussion

We propose elementary differential geometry and texturization methods to inspect 3*D* medical volumes arising from MRI and CT studies. The method is based on fitting surfaces to the object of interest and common computer graphics texturization techniques.

We consider three examples

- rotator cuff tear visualization,
- abdominal aortic aneurysm display along transversal curved slices and
- odontological visualization.

The examples pertain to very different fields of medical healthcare which, from the visualization perspective, are treated in a fairly unified way, using parametric surfaces placed within a medical volume. The techniques are applied to the most common medical volumes: MRI and CT, but in principle may be applied to any volumes, gathered with any methodology. The visualization technique is based on two elements: choosing the surface in the area of interest which entails a fitting process and the texturization of this surface. The data points of the volume do not have to be organized in any structured way. The nearest neighbor technique delivers faithful texturizations of parametric surfaces for any reasonable data point distributions.

The technique can be extended to volumetric inspection by moving the texturized surface within the medical volume. This proves useful to produce animations (see the rotator cuff animation in the Additional file 1 of the web version of this paper). The technique also offers visualization of the morphology along the intersection of transversal surfaces, as illustrated in the case of aortic abdominal aneurysms and along the root canal of a dental piece. In the case of aortic aneurysms and the jaw, the curvilinear surfaces may be chosen to be developable, which allows for their flat display without deformation.

In the case of the rotator cuff we illustrate the technique by taking a sequence of offsets, i.e. of “parallel” fitting surfaces which allows for the localization of possible tears within the 3*D* volume. The research shows that it is possible to offer high quality visualization without the need of specialized software.

It is not difficult to envision other applications in medical visualization such as vascular calcifications and implantology in dentistry, among others.

Finally we would like to bring forward the idea that texturized curved slices can be used as navigation devices within medical volumes. Moreover, since are given by parametric surfaces their shape can be changed as well as their position. In the particular case that the surface is developable it may be flattened without distortion.

The main advantage of texturized curved slices is lower cost as compared volumetric rendering of organs, flexibility (shape change) and adaptability (position change).

## Conclusions

The inspection of medical volumes given by the texturization of surfaces adapted to the geometry of the object of interest is possible using surface techniques of differential geometry and texturization processes of common use in computer graphics. The technique was illustrated in various MRI and CT examples, including the inspection of rotator cuff injuries, aortic aneurysms and in dental visualization. Since the technique is relatively computationally inexpensive it should play a role in the clinical practice, especially in low to medium income communities. More interdisciplinary efforts will be necessary to bring such a possibility to fruition.

## Data Availability

The sources of the datasets used in this study are as follows: the abdominal aortic aneurysm data were pubicly available in the world wide web, the rotator cuff and the odontological datasets are from the personal library of M. Paluszny. The datasets are available from the authors upon request.

## Abbreviations

CT: computed tomography
DICOM: Digital imaging and communications in medicine
MRI: magnetic resonance imaging

## Footnotes

1 Osirix DICOM viwer - http://www.osirix-viewer.com/resources/dicom-image-library/for Mac OS and RadiAnt - https://idoimaging.com/programs/107, for Windows.

2 Each slice is presented as a two dimensional image which is built as a matrix (usually with fairly high resolution pxq, where p and q are a few hundred). Moreover, very frequently, for individual imaging studies, only a fraction of the gathered information is used.

3 In Matlab the access to the header is provided by the command dicominfo. It requires the Image Processing Toolbox.

4 In most cases, as illustrated in Fig. 3, the box coincides with the volume occupied by the slices in 3D. But it is not always so as is exemplified in Fig. 5.

5 In $\mathbf {a}_{_{FL}}$*F* and *L* refer to front and lower (similarly *B* and *R*), the **a** vertices lie in one face and the **b** vertices correspond to its parallel face.

6 As stated above, the information regarding the positions of the planes and the positions of the points may be computed from the information in the fields ImagePositionPatient, ImageOrientationPatient and PixelSpacing in the headers.

7 It may happen that some sub-boxes do not contain data points, so for a point in the discretization of the curved slice which lies in this sub-box no gray level is assigned.

8 The fitting ellipsoid consists of circles that lie on parallel planes and their centers live on an orthogonal line to these planes. An offset surface consists of circles with the same centers and their radii are increased or decreased by a small fixed number.

9 The fitted ellipsoid mostly contains the humerus bone head.

10 The book contains engaging illustrations of various types of root canal systems which are usually hard to treat.

11 Mathematically, the 1:1 mapping between a developable surface and its flat image is referred to as an isometry.

12
*G*^1^ refers to geometrically continuous joining of curves or surfaces.

13 In general, a developable surface is a ruled surface such that the tangent plane is the same along each ruling.

14 The geodesic curvature of curve on a surface measures how much it deviates from a geodesic, i.e. a curve that minimizes distance.

15 The curvature of a planar curve measures how much it deviates from a straight line.

## References

[1] Fuchs H, Levoy M, Pizer S (1989). Interactive visualization of 3*d* medical data. Computer.

[2] Zhang Q, Eagleson R, Peters TM (2011). Volume visualization: a technical overview with focus on medical applications. J Digit Imaging.

[3] Wheeler G, Deng S, Toussaint N, Pushparajah K, Schnabel J, Simpson J, Gomez A (2018). Virtual interaction and visualization of 3*d* medical imaging data with VTK and Unity. Health Care Technol Lett.

[4] Smith-Bindman R, Miglioretti DL, Johnson E, Lee C, Feigelson HS, Flynn M, Greenlee RT, Kruger RL, Hornbrook MC, Roblin D (2012). Use of diagnostic imaging studies and associated radiation exposure for patients enrolled in large integrated health care systems, 1996–2010. Jama.

[5] Preti G, Pera P, Bassi F (1986). Prediction of the shape and size of the maxillary anterior arch in edentulous patients. J Oral Rehabil.

[6] Figueiredo O, Hersch RD (2002). Parallel unfolding and visualization of curved surfaces extracted from large 3D volumes. J Electron Imaging.

[7] González C, Pérez MI, Lentini M, Ríos D, Albrecht G, Paluszny M (2017) Dental information along curved slices In: Computing Conference, 1410–1413.. IEEE. 10.1109/sai.2017.8252279.

[8] González C, Paluszny M (2015). Odontological information along cone splines. Analysis, Modelling, Optimization, and Numerical Techniques.

[9] Gibbs J, Petty DD, Robins N, et al. (2002) Painting and rendering textures on unparameterized models In: ACM Transactions on Graphics (TOG), vol. 21, 763–768.. ACM. 10.1145/566570.566649.

[10] Buss SR (2003). 3-D Computer Graphics: a Mathematical Introduction with OpenGL.

[11] Bourke P (1999) Trilinear Interpolation. http://paulbourke.net/miscellaneous/interpolation/. Accessed 19 Jan 2020.

[12] Peters OA (2016). The Guidebook to Molar Endodontics.

[13] Petersson A, Axelsson S, Davidson T, Frisk F, Hakeberg M, Kvist T, Norlund A, Mejàre I, Portenier I, Sandberg H (2012). Radiological diagnosis of periapical bone tissue lesions in endodontics: a systematic review. Int Endod J.

[14] Vrtovec T, Likar B, Pernus F (2005) Curved planar reformation of CT spine data In: Medical Imaging 2005: Image Processing, vol. 5747, 1446–1457.. International Society for Optics and Photonics. 10.1117/12.595113.

[15] Paluszny M (2011) Between developable surfaces and circular cone splines: curved slices of 3D volumes In: Medical Imaging 2011: Visualization, Image-Guided Procedures, and Modeling, vol. 7964, 796435.. International Society for Optics and Photonics. 10.1117/12.872157. Accessed 19 Jan 2020.

[16] Aumann G (2003). A simple algorithm for designing developable Bézier surfaces. Comput Aided Geom Des.

[17] Fuhs W, Stachel H (1988). Circular pipe-connections. Comput Graph.

[18] Paqué F, Balmer M, Attin T, Peters OA (2010). Preparation of oval-shaped root canals in mandibular molars using nickel-titanium rotary instruments: a micro-computed tomography study. Journal of Endodontics.

[19] Peters OA, Paqué F (2011). Root canal preparation of maxillary molars with the self-adjusting file: a micro-computed tomography study. J Endod.

[20] Karabucak B, Bunes A, Chehoud C, Kohli MR, Setzer F (2016). Prevalence of apical periodontitis in endodontically treated premolars and molars with untreated canal: a cone-beam computed tomography study. J Endod.

[21] Woo J, Greene CManagement of thoracic aortic aneurysm in adults. https://www.uptodate.com/contents/management-of-thoracic-aortic-aneurysm-in-adults. Accessed 19 Jan 2020.

[22] Saliba E, Sia Y, Dore A, El Hamamsy I (2015). The ascending aortic aneurysm: When to intervene?. IJC Heart Vasculature.

[23] Alam F, Rahman SU, Rahman AU (2018) An algorithm for extracting centerline of the aorta from CT/MR 3*D* images In: 2018 6th International Conference on Biological and Medical Sciences (ICBMS 2018), 176–180.. Seoul National University. https://dl.acm.org/doi/10.1145/3278229.3278234. Accessed 19 Jan 2020.

[24] Do Carmo MP (1976). Differential Geometry of Curves and Surfaces.

[25] Pottmann H, Wallner J (2009). Computational Line Geometry.

